# Changes in Cytokeratin 18 during Neoadjuvant Chemotherapy of Breast Cancer: A Prospective Study

**DOI:** 10.30699/ijp.2020.116238.2261

**Published:** 2020-02-19

**Authors:** Danial Fazilat-Panah, Somaye Vakili Ahrari Roudi, Alireza Keramati, Azar Fanipakdel, Mohammad Hadi Sadeghian, Fatemeh Homaei Shandiz, Soodabeh Shahidsales, Seyed Alireza Javadinia

**Affiliations:** 1 *Cancer Research Center, Babol University of Medical Sciences, Babol, Iran*; 2 *Department of Pathology, Student Research Committee, Mashhad University of Medical Sciences, Mashhad, Iran*; 3 *Department of Radio-Oncology, Student Research Committee, Mashhad University of Medical Sciences, Mashhad, Iran*; 4 *Cancer Research Center, Mashhad University of Medical Sciences, Mashhad, Iran*; 5 *Department of Pathology, Student Research Committee, Mashhad University of Medical Sciences, Mashhad, Iran*; 6 *Cellular and Molecular Research Center, Sabzevar University of Medical Sciences, Sabzevar, Iran*

**Keywords:** Breast carcinoma, Neoadjuvant therapy, Cytokeratin-18, M30 cytokeratin-18 peptide, M65 cytokeratin-18 peptide

## Abstract

**Background & Objective:**

Prediction of response to neoadjuvant treatment is an important part of treatment of patients with breast cancer. This study aimed to assess changes in serum levels of Cytokeratin 18 during neoadjuvant chemotherapy in patients with locally advanced breast cancer and its association with neoadjuvant treatments.

**Methods:**

This research was performed on newly diagnosed breast cancer patients referred to Omid Radiotherapy Center and radiotherapy and oncology departments of Emam Reza and Ghaem hospitals, in Mashhad, Iran. Serum levels of M30 and M65 fragments of Cytokeratin 18 were measured before and 24 hours after the first course of neoadjuvant chemotherapy. Changes in serum levels of Cytokeratin 18 and its fragments and their correlation with pathologic response were analyzed.

**Results:**

Pre- and post-chemotherapy levels of M30 were respectively 223.9±18.94 and 250.7±23.92 U/L (*P*=0.24). For M65, these levels were respectively 301.5±313.9 and 330.2±352.2 U/L (*P*=0.1). Changes in M30 level during chemotherapy in patients with and without pathologic complete response were -20±92.69 and 43.1±106.5, respectively (*P*=0.1). For M65, these changes were respectively -247±55 and 76±240 (*P*=0.1). Baseline levels of M30 and M65 had no relation with menopausal status, tumor grade, hormone receptor status, Ki67 expression, molecular subtype, and stage.

**Conclusion:**

Our findings showed statistically insignificant changes in the level of Caspase-cleaved- (M30) and uncleaved- (M65) cytokeratin 18 fragments (apoptotic and necrotic indicators, respectively) during neoadjuvant chemotherapy in patients with breast cancer. There was no notable relationship between tumor-related factors and either baseline levels or serum changes of CK18 fragments. Also, there was no correlation between M30/M65 level and pathologic response to neoadjuvant chemotherapy.

## Introduction

Breast cancer is one of the most commonly diagnosed neoplasms in women (1,2). In recent decades, remarkable progress in systemic chemotherapy, hormone therapy, and targeted therapy has significantly reduced the mortality of stage I, II, and III breast cancer patients. Given the benefits observed in adjuvant treatments, recent years has seen growing interest in the use of neoadjuvant treatments for earlier inhibition of micrometastasis in order to improve the treatment outcome (3). Today, neoadjuvant chemotherapy treatment is widely accepted as a standard treatment for locally advanced and inflammatory breast cancers. Research has shown that patients whose primary tumor exhibits a pathologic complete response (pCR) to neoadjuvant treatment generally have better treatment outcomes and survival rates (4).

Assessment of response to neoadjuvant treatment is of paramount importance for preventing excess toxicity in patients who do not benefit from chemotherapy. For most patients, this assessment only involves clinical examination or imaging to track changes in the primary tumor and adjacent lymph nodes (5). Currently, there is no standard method for assessment of the response to neoadjuvant chemotherapy in breast cancer patients; however, several studies have shown that mammography, ultrasound, and MRI examinations may have a role in the prediction of pCR (5-8). However, there is still no standard protocol for a definitive diagnosis of the response of primary tumors to neoadjuvant chemotherapy.

Cytokeratins are among the most important components of the epithelial cell structure and have unique expressions in different epithelial cells. Two important cytokeratins of the intermediate filament family are cytokeratin 18 and cytokeratin 8, which belong to the group of type I and type II cytokeratins respectively. Together, these cytokeratins play a notable role in important cellular processes such as proliferation and cellular mitosis. During apoptosis and necrosis processes, caspase-cleaved- (M30) and uncleaved- (M65) cytokeratin 18 fragments appear at an elevated level in blood serum. Therefore, serum levels of these fragments can perhaps be used as a marker for diagnosis and to predict prognosis or response to treatment (9-13).

In a study by Stoetzer et al., the possibility of using the level of cytokeratin 8/18 fragments (M30 level) as a specific marker for apoptosis in healthy subjects (146 U/L), patients with benign breast tumors (131 U/L), patients with local breast cancer (165 U/L), and patients with metastatic breast cancer (198 U/L) was studied. Ultimately, this study reported that only the M30 level of metastatic patients was significantly higher than that of healthy individuals (12). In a study carried out by Demiray et al., where the monoclonal level of M30 antibody was measured as the representative of cytokeratin 8/18 fragment, the M30 levels of breast cancer patients 24 hours (100/5 U/L) and 48 hours (86/5 U/L) after neoadjuvant chemotherapy were significantly higher than the baseline level (64/9 U/L) (9). Also, the change in M30 level 24 hours after chemotherapy was significantly higher in responders (increase in median M30 level from 63 U/L to 79 U/L with P=0.001) than in non-responders (increase in median M30 level from 80 U/L to 105 U/L with P= 0.05). However, a study by Fehm et al. reported that although the pathologic response of breast cancer patients could be predicted by apoptotic DTC (in the sense that none of the patients with progressing disease had rising M30 level), some of patients with a pathologic complete response (and elevated marker level) could still have non-apoptotic cells and therefore benefit from secondary adjuvant chemotherapy (10). A study by Tas et al. reported that although the serum M30 level was higher in metastatic patients, it was not significantly related to the prognosis and survival. This study also found no correlation between the serum M30 level and the response to chemotherapy (13).

The present study aimed to investigate the relationship between baseline level and serum changes of cytokeratin 18 fragments M30 and M65 before and after neoadjuvant chemotherapy with pathologic response in breast cancer treatment.

## Materials and Methods

This cross-sectional study was carried out from 2015 to 2017 on newly diagnosed breast cancer patients who were eligible to receive neoadjuvant chemotherapy. Subjects were selected by convenient sampling from the patients referred to Omid Hospital and RadioOncology department of Emam Reza and Ghaem hospitals, all three affiliated with Mashhad University of Medical Sciences. Inclusion criteria were the diagnosis of inflammatory breast cancer, diagnosis of locally advanced breast cancer (T4 or N2-3), and or diagnosis of large tumors with patient preferring to undergo breast-conserving surgery. Exclusion criteria were the refusal to enroll or continue participating in the study and the presence of distant metastasis at the time of enrollment. The research protocol was pre-confirmed by the Ethics Committee of Mashhad University of Medical Sciences and all participating patients signed the form of informed consent at enrollment.

The research was performed through the following procedure. Venous blood samples of 5cc in volume were collected before and 24 hours after chemotherapy. The samples were centrifuged at 2200-2500 RPM for 15 minutes immediately after sampling and then stored at 20°C until the test of CK18 level. To measure the post-chemotherapy level of apoptosis and necrosis in ductal carcinoma cells, the serum levels of the antibodies bound to caspase-cleaved CK18 and uncleaved CK18 were measured using the M30 Apoptosense® CK18 Kit  (DiaPharma®, USA) and  M65 EpiDeath® ELISA kit (DiaPharma®, USA) respectively. These kits are solid-phase sandwich enzyme immunoassays, where samples, a positive control, and a standard control react with a solid phase capture antibody M5 directed against CK18 and the HRP-conjugated M30 antibody directed against CK18Asp396 neo-epitope (for M30) or the HRP-conjugated M6 antibody (for M65). In these assays, unbound conjugates are removed by washing and 3,3′,5,5′-Tetramethylbenzidine substrate is added, and then the color development is stopped and the absorbance is read. The intensity of the color is proportional to the concentration of the antigen. Finally, the assays give the antigen concentration in units per liter (U/L). For these assays, first, M30 or M65 conjugates were diluted by adding 9.2 mL of buffer to the vials containing exactly 0.4 mL of M30 or M65 conjugate. Next, 25 μL of control solution (Control Low and Control High), standard solution, or patient sample were injected into the wells with a pipette. Each well was then injected with 75 μL of the diluted M30 or M65 Conjugate solution. The wells were sealed with glue and incubated for 4 hours at 600 RPM in a shaker. The sample container was then washed five times with 400-500 μL/well of a solution prepared by dissolving a Wash Tablet in 500 mL of deionized water. After adding 200 μL of the TMB substrate to each well, the samples were incubated for 20 minutes in dark at room temperature. After these 20 minutes, each well was injected with 50 μL of Stop Solution, shaken for 5 to 10 seconds, and then left to rest for five minutes at room temperature. Finally, the absorbance was measured by a microplate reader at 450 nm. The concentration was calculated from the absorbance using the Cubic Spline curve of absorbance at 450 nm (A450) versus concentration (U/L).

All epidemiologic factors and associated clinical and pathologic factors were recorded. Patients underwent surgery 2-3 weeks after the completion of neoadjuvant chemotherapy. After the determination of pathologic response, the relationship of the serum levels of CK18 after neoadjuvant chemotherapy with the postoperative pathologic response was analyzed. The pathologic complete response was defined as the absence of tumor cells in the breast tissue and axillary lymph nodes. The cases where tumor cells remained in breast tissue or lymph nodes were considered as the pathologic incomplete response.

The collected data were entered into SPSS 21 (SPSS Inc. Chicago, Ill., USA) and GraphPad Prism v7 for descriptive and inferential analysis. For descriptive data, frequency, percentage, mean, and standard deviation were computed. Before each analysis, the Kolmogorov-Smirnov test was performed to check the normality of data and determine whether parametric or nonparametric methods should be used. In the case of normal distribution, the means were compared using independent T or One-way ANOVA tests. Otherwise, the non-parametric Mann-Whitney test was used for this comparison. All tests were performed at P-value≤0.05 significance level.

## Results

**Fig. 1:**
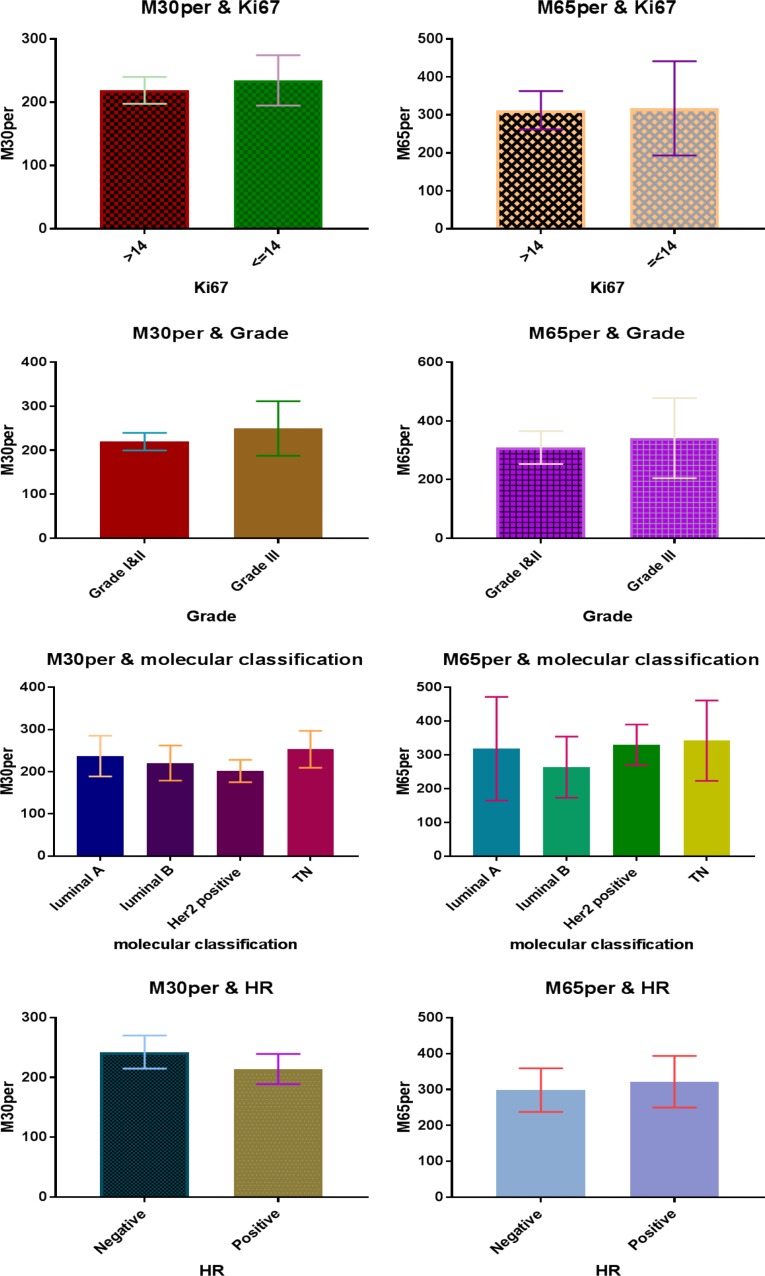
Relationship of baseline M30 and M65 levels with tumor characteristics

**Fig. 2:**
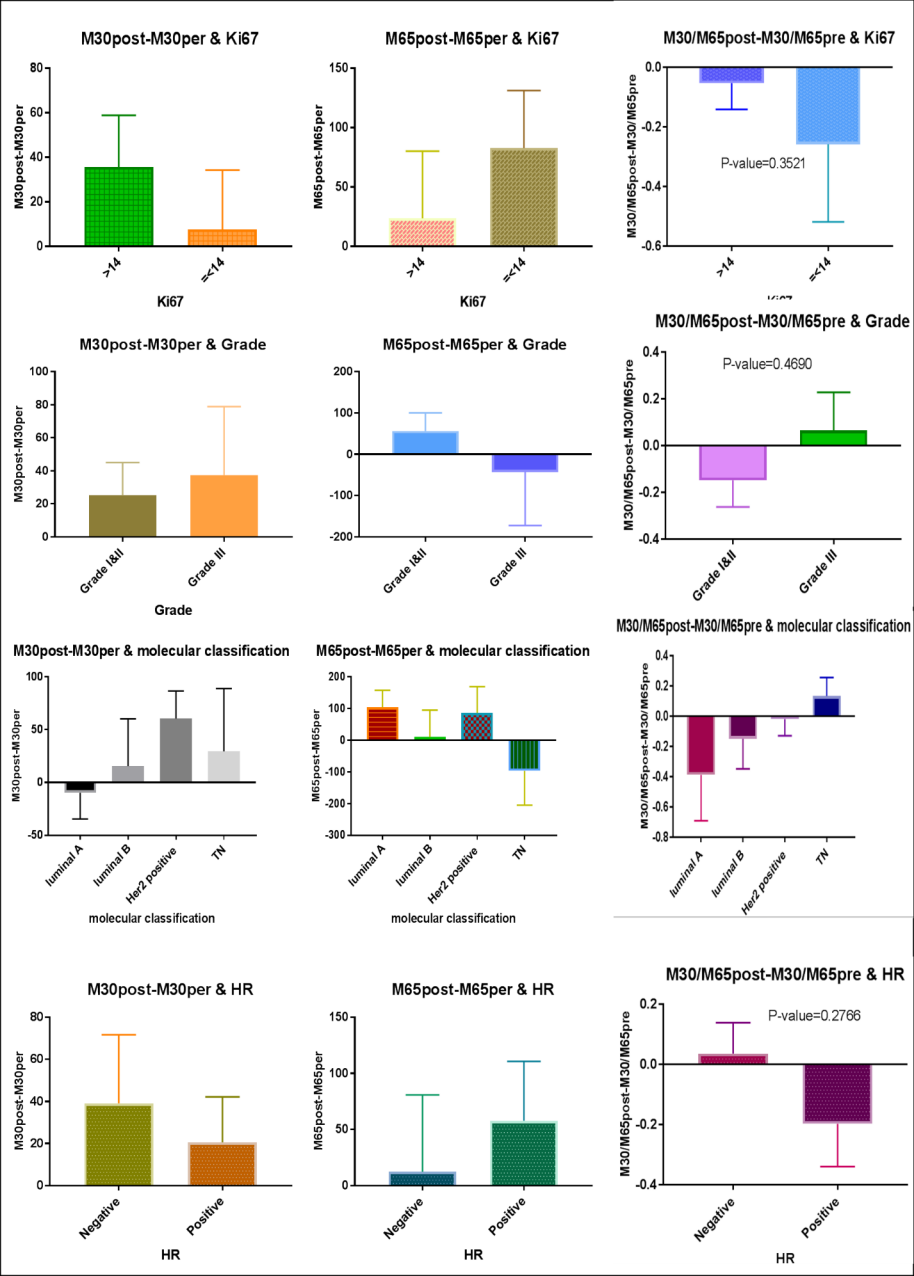
Relationship of changes in M30 and M65 levels with tumor characteristics

A total of 35 patients entered the study. The mean age of the patients was 46.17±10 years. Most of the patients were pre-menopausal (22 cases, 62.9%). The basic information of the patients is provided in Table 1. No correlation was observed between tumor characteristics and M30 and M65 levels and their changes during chemotherapy (Figures 1 and Fig2).

**Fig. 3:**
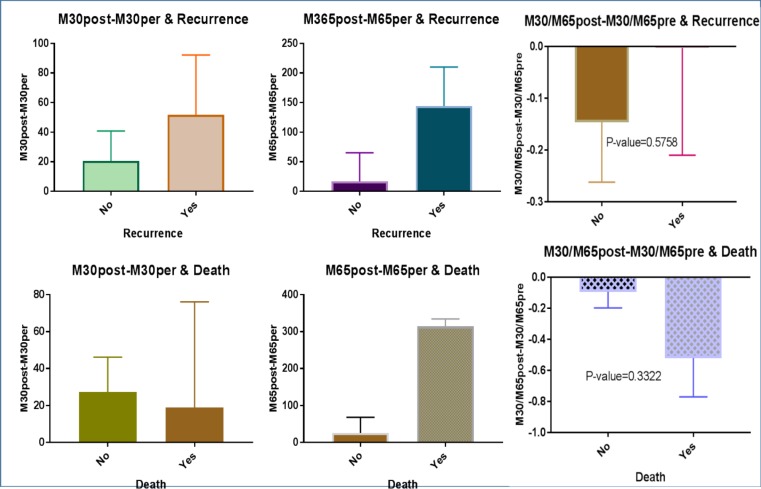
Relationship of changes in M30 and M65 levels with survival

 The pre- and post-treatment serum levels of M30 were respectively 223.9±18.94 and 250.7±23.92 U/L (*P*=0.24). For M65, the serum levels before and after treatment were respectively 313.9±50.96 and 356.2±55.82 U/L (*P*=0.1). The pre- and post-treatment M30/M65 ratio was respectively -1.07±0.14 and -0.95±0.10 (*P*=0.4). No relationship was found between changes in these tumor markers and survival statistics (Figure 3). The mean change in the M30 level for the patients with pathologic complete response to chemotherapy (n=9) was -20±92.69, and for the patients without complete response (n=26), it was 70.06±240 (*P*=0.2) (Figure 3).

**Table 1:** Characteristics of subjects in the control and intervention groups (at the time of enrollment)

		Group	
Variable		Frequency	Percentage
**Age (years)**	≤40	11	31.4
	>40	24	68.6
**Menstrual Status**	Pre-menopause	22	62.9
	Post-menopause	13	37.1
**Clinical T**	T1	2	5.7
	T2	15	42.9
	T3	10	28.6
	T4	8	22.9
**Clinical N**	N1	14	40
	N2	10	28.6
	N3	3	8.6
	Unknown	8	22.9
**Tumor Grade**	Grade I	6	17.1
	Grade II	24	68.6
	Grade III	5	14.3
**ER Status**	Negative	12	34.3
	Positive	23	65.7
**PR Status**	Negative	17	48.6
	Positive	18	51.4
**Hormone Receptor Status**	Negative	12	34.3
	Positive	23	65.7
**HER2 Status**	Negative	23	65.7
	Positive	12	34.3
**Ki67 Status**	≤14%	11	31.4
	>14%	24	68.6
**Molecular Subtype**	Luminal A	9	25.7
	Luminal B	8	22.9
	HER2-enriched	12	34.3
	triple negative	6	17.1

**Table 2:** General information of some of the studies performed on different aspects of cytokeratin 18 in breast cancer patients

No.	First Author	Year	Country	Sample size	Method	Measurement Type
1	Mutlu Demiray (9)	2006	Turkey	42	ELISA	Serum
2	Tanja Fehm (10)	2006	Germany	157	IHC	BM
3	Hägg Olofsson (20)	2007	Japan	61	ELISA	Serum
4	Engin Ulukaya (22)	2011	Turkey	37	ELISA	Serum
5	Oliver Stoetzer (12)	2013	Germany	79	ELISA	Serum
6	Faruk Tas (13)	2014	Turkey	80	ELISA	Serum
7	Natalia Krawczyk (23)	2014	Germany	298	IHC	Breast Tissue/BM
8	Gerhard Schaller (24)	1996	Germany	43	IHC	Breast Tissue
9	Ute Woelfle (25)	2004	Germany	1458	IHC	Breast Tissue
10	Maria Hagg Olofssion (20)	2007	Japan	45	ELISA	Serum
11	Faruk Tas (13)	2014	Turkey	80	ELISA	Serum
BM: bone marrow, IHC: immunohistochemistry						

## Discussion

 Breast cancer is the most commonly occurring cancer in women. The biomarkers traditionally used in predicting the prognosis of breast cancer and its response to treatment include the expression of estrogen and progesterone receptors and the rate of HER2 gene amplification. Given that each molecular subtype of breast cancer itself consists of several heterogeneous sub-branches, there is a growing need to discover new biomarkers for predicting the most effective treatment approaches. Since personalized medicine and neoadjuvant chemotherapy have become increasingly popular treatment modalities in different malignancies, it is important to find a marker for early identification of those patients who will benefit from this approach (14-16). Response to neoadjuvant treatments may be predicted by cell death markers such as cytokeratins (17-19). This study investigated the relationship of serum CK18 levels before and after neoadjuvant chemotherapy with pathologic response to breast cancer.

The results showed that the patients with tumors of higher grades had higher pre-treatment M30 and M65 levels. The hormone-receptor-negative patients had higher serum M30 levels and the hormone-receptor-positive patients had higher serum M65 levels. The patients with a higher level of proliferation and Ki67 expression were found to have a higher level of M30, but in contrast, when Ki67 expression was lower, the M65 level was higher. The assessment of M30 level in different molecular subtypes showed that before treatment the patients with triple negative breast cancer had the highest level of M30 and the patients with luminal A cancer had the highest level of M65. Note that none of the reported differences was statistically significant. Overall, the results showed that, after chemotherapy, the serum levels of M30 and M65 increased but the M30/M65 ratio decreased. However, none of these changes was statistically significant.

In the assessment of the relationship of changes in M30 and M65 levels with the pathologic response to neoadjuvant chemotherapy, it was found that the patients with a pathologic complete response had lower M30, M65, M30/M65 levels after treatment, but other patients had higher levels of M30, M65, M30/M65 after treatment.

The following is a report of M30 serum levels based on patient and tumor-related factors. The greatest changes in serum M30 level was observed in the patients with higher grade tumors, hormone-receptor-negative subtype, higher Ki67 levels, HER2-enriched subtype, and triple negative subtype. However, none of these changes was statistically significant at 0.05 level. Regarding M65, the greatest change in serum levels was related to the patients with higher grade tumors, hormone-receptor positive, lower Ki67 levels, luminal B subtype, and triple negative subtype. But again, none of these changes was statistically significant at 0.05 level. Finally, the relationship of changes in M30 and M65 levels with survival and recurrence was examined. This examination showed that the patients who experienced recurrence had greater changes in M30 and M65 levels and M30/M65 ratio than disease-free patients. In contrast, changes in M30 were higher in surviving patients and changes in M65 and M30/M65 were higher in non-surviving patients. However, none of these differences was statistically significant.

Table 2 shows the general information of some of the studies performed on different aspects of cytokeratin 18 in breast cancer patients. Most of the studies in this area have measured the changes in M30 level of breast cancer patients and the studies on the changes in M65 or M30/M65 ratio are less frequent.

In the present study, the results showed increased serum M30 and M65 levels after neoadjuvant chemotherapy, but the observed changes were not statistically significant. This is consistent with the majority of existing reports in this area, although there has been some discrepancy in the exact change in serum levels of this marker following chemotherapy treatment. For example, it has been reported that for breast cancer patients, docetaxel-containing treatments are associated with a significant increase in the serum level of M30, but treatment with cyclo-phosphamide/epirubicin/5FU results in heterogeneous responses are often accompanied with increased M65 levels (20). Given that the M30 subunit of cytokeratin 18 is released after apoptosis and M65 is released after necrosis, the serum levels of these markers can be expected to increase following chemotherapy. Similar findings have been reported by the studies of Ulukaya *et al.* (2015), Gemechu *et al.* (2018) and Demiray *et al.* (2006) (9,21,22). In the study conducted by Ulukaya *et al.*, assessment of changes in the cleaved subunit of cytokeratin 18 during neoadjuvant chemotherapy in breast cancer patients showed significantly increased serum levels 24 hours after the initiation of treatment (22). Similarly, the study carried out by Gemechu *et al.* reported an elevated serum level of M30 (as the cleaved fragment of cytokeratin 18) six hours after chemotherapy (21). The study conducted by Demiray *et al.* also reported that the cleaved fragment of cytokeratin 18 increased after neoadjuvant chemotherapy (9). An interesting finding in the Demiray’s study was the decline of this marker 48 hours after the treatment from the peak serum level observed within the first 24 hours, which highlights the importance of measurement time for the assessment of this marker.

This study found no statistically significant relationship between changes in the serum levels of M30 and M65 and the pathologic response of breast cancer to neoadjuvant chemotherapy. Similarly, the studies performed by Stoetzer *et al.* and Tas *et al.* also found no relationship between pathologic response and serum levels of M30 or M65 (12,13). The study conducted by Stoetzer *et al.*, that examined the response prediction power of several apoptotic markers in the serum samples of breast cancer patients (including M30), found no significant difference in the serum level of M30 in patients with and without response to neoadjuvant chemotherapy (12). Tas *et al.*, who examined the serum levels of M30 and M65 before and after neoadjuvant chemotherapy in 80 breast cancer patients, also reported that this marker has no potential value for predicting the tumor response to chemotherapy (13). In contrast, studies carried out by Demiray *et al.* and Olofsson *et al.* among others have reported a significant change in these markers following the neoadjuvant chemotherapy (9,20). Demiray’s study, which was conducted on 42 breast cancer patients undergoing neoadjuvant chemotherapy with anthracycline, found a significant relationship between changes in M30 level and the overall tumor response to neoadjuvant chemotherapy, in the sense that patients whose tumors responded to chemotherapy had significantly increased M30 levels. However, it should be noted that the patients whose tumors did not respond to chemotherapy also showed an insignificant increase in this variable (9). The study performed by Olofsson (2007), which used cytokeratin 18 to monitor the cell death induced by each course of chemotherapy with docetaxel or ECF in 61 breast cancer patients, reported a significant relationship between increased serum M30 level and the pathologic response to these chemotherapies (20).

Concerning the relationship between serum M30 and M65 levels and clinicopathologic factors, the results of this study showed higher M30 and M65 levels in patients with higher grade tumors, triple negativity, and more node involvement. Also, higher M30 levels were observed in patients with hormone-receptor-negative subtype and higher proliferation, and higher M65 levels were seen in patients with hormone-receptor-positive subtype and lower proliferation. However, the observed differences were not statistically significant. Previous studies have reported contradictory results in this regard. In the research performed by Tas *et al.*, the results indicated higher serum M30 and M65 levels in patients with hormone-receptor-negative subtype, HER2 negative subtype, and higher grade tumors (13). The results of Demiray *et al.* showed a significant increase in the serum M30 level of patients with Grade III tumors. However, there was no significant difference in serum levels of this marker based on hormone receptor or other tumor characteristics (9). The results of Tas *et al.*, Demiray *et al.* and this study are however inconsistent with the findings of a research by Krawczyk *et al.*, where the incidence of M30 in breast tumor tissue was investigated by immunohistochemistry and the results showed higher incidence of this cytokeratin in hormone-positive and HER2-positive subtypes and cancers with lower grade tumors (23).

The results of the present study showed no significant relationship between changes in M30, M65 and M30/M65 ratio during chemotherapy and survival parameters. Other studies have also reported that the serum levels of M30 and M65 and their changes during neoadjuvant chemotherapy have no prognostic value for predicting survival in patients with breast cancer (13).

The most important limitation of this study was the small size and high heterogeneity of the sample studied. This heterogeneity was present not only in tumor biology but also in patients’ therapeutic regimens (24).

Future studies are therefore suggested to use a larger sample of breast cancer patients with higher homogeneity in terms of molecular criteria. It is also recommended to investigate the impact of other interventions such as surgery and bulk tumor removal on the serum level of the studied markers. A longer assessment of this serum marker during neoadjuvant chemotherapy (repeating measurements 48 and 72 hours after treatment and after each course of chemotherapy) may also provide a better insight into the physiology of M30 and M65 (25).

## Conclusion

This study found statistically insignificant changes in serum M30 and M65 levels (cleaved and uncleaved fragments of cytokeratin 18) following neoadjuvant chemotherapy in breast cancer patients. No relationship was observed between the baseline level of this marker before treatment and tumor characteristics, which indicates the lack of prognostic value. There was also no relationship between changes in serum M30 and M65 levels and pathologic response of breast tumors to chemotherapy. According to these findings, M30 and M65 cannot serve as accurate markers for predicting breast cancer response to neoadjuvant chemotherapy.

## References

[1] Jemal A, Bray F, Center MM, Ferlay J, Ward E, Forman D (2011). Global cancer statistics. CA: a cancer journal for clinicians..

[2] Salek R, Shahidsales S, Mozafari V (2016). Changing pattern in the clinical presentation of breast cancer in the absence of a screening program over a period of thirty-three years in Iran. The Breast..

[3] Mamounas EP, Fisher B (2001). Preoperative (neoadjuvant) chemotherapy in patients with breast cancer. Semin Oncol..

[4] Cardoso F, Senkus E, Costa A, Papadopoulos E, Aapro M, André F (2018). 4th ESO-ESMO International Consensus Guidelines for Advanced Breast Cancer (ABC 4)†. Annals of Oncology..

[5] Teshome M, Hunt KK (2014). Neoadjuvant therapy in the treatment of breast cancer. Surg Oncol Clin N Am..

[6] Kaufmann M, von Minckwitz G, Mamounas EP (2012). Recommendations from an international consensus conference on the current status and future of neoadjuvant systemic therapy in primary breast cancer. Ann Surg Oncol..

[7] Peintinger F, Kuerer HM, Anderson K, Boughey JC, Meric-Bernstam F, Singletary SE (2006). Accuracy of the combination of mammography and sonography in predicting tumor response in breast cancer patients after neoadjuvant chemotherapy. Annals of surgical oncology..

[8] Rauch GM, Adrada BE, Kuerer HM, van la Parra RFD, Leung JWT, Yang WT (2017). Multimodality Imaging for Evaluating Response to Neoadjuvant Chemotherapy in Breast Cancer. American Journal of Roentgenology..

[9] Demiray M, Ulukaya EE, Arslan M, Gokgoz S, Saraydaroglu O, Ercan I (2006). Response to neoadjuvant chemotherapy in breast cancer could be predictable by measuring a novel serum apoptosis product, caspase-cleaved cytokeratin 18: a prospective pilot study. Cancer investigation..

[10] Fehm T, Becker S, Becker-Pergola G, Sotlar K, Gebauer G, Durr-Storzer S (2006). Presence of apoptotic and nonapoptotic disseminated tumor cells reflects the response to neoadjuvant systemic therapy in breast cancer. Breast Cancer Res..

[11] Oshima RG, Baribault H, Caulin C (1996). Oncogenic regulation and function of keratins 8 and 18. Cancer Metastasis Rev..

[12] Stoetzer OJ, Fersching DMI, Salat C, Steinkohl O, Gabka CJ, Hamann U (2013). Prediction of response to neoadjuvant chemotherapy in breast cancer patients by circulating apoptotic biomarkers nucleosomes, DNAse, cytokeratin-18 fragments and survivin. Cancer Letters..

[13] Tas F, Karabulut S, Yildiz I, Duranyildiz D (2014). Clinical significance of serum M30 and M65 levels in patients with breast cancer. Biomedicine & Pharmacotherapy..

[14] Javadinia SA, Shahidsales S, Fanipakdel A, Mostafapour A, Joudi‐Mashhad M, Ferns GA (2018). The Esophageal Cancer and the PI3K/AKT/mTOR Signaling Regulatory microRNAs: a Novel Marker for Prognosis, and a Possible Target for Immunotherapy. Current Pharmaceutical Design..

[15] Javadinia SA, Shahidsales S, Fanipakdel A, Joudi‐Mashhad M, Mehramiz M, Talebian S (2019). Therapeutic potential of targeting the Wnt/β‐catenin pathway in the treatment of pancreatic cancer. Journal of Cellular Biochemistry..

[16] Fanipakdel A, Seilanian Toussi M, Rezazadeh F, Mohamadian Roshan N, Javadinia SA (2019). Overexpression of cancer-testis antigen melanoma-associated antigen A1 in lung cancer: A novel biomarker for prognosis, and a possible target for immunotherapy. Journal of Cellular Physiology..

[17] Fanipakdel A, Javadinia S, Memar B, Homayundust S (2019). P-008 Plasma cytokeratin-18 level of patients with esophagogastric cancer as a biomarker of tumour response. Annals of Oncology..

[18] Turk HM, Aliyev A, Celik RS, Seker M, Coban E, Demir T (2020). Usefulness of serum M30 and M65 levels to predict response to neoadjuvant chemotherapy in patients with breast cancer. Current Problems in Cancer..

[19] Abdelrahman AE, Rashed HE, Abdelgawad M, Abdelhamid MI (2017). Prognostic impact of EGFR and cytokeratin 5/6 immunohistochemical expression in triple-negative breast cancer. Annals of Diagnostic Pathology..

[20] Olofsson MH, Ueno T, Pan Y, Xu R, Cai F, van der Kuip H (2007). Cytokeratin-18 is a useful serum biomarker for early determination of response of breast carcinomas to chemotherapy. Clinical Cancer Research..

[21] Gemechu Y, Seifu D, Tigneh W, Labisso WL (2018). Caspase-Cleaved Cytokeratin 18 as a Potential Molecular Biomarker for Monitoring Chemotherapeutic Response in Breast Cancer Patients. J Mol Genet Med.

[22] Ulukaya E, Yilmaztepe A, Akgoz S, Linder S, Karadag M (2007). The levels of caspase-cleaved cytokeratin 18 are elevated in serum from patients with lung cancer and helpful to predict the survival. Lung Cancer..

[23] Krawczyk N, Hartkopf A, Banys M, Meier-Stiegen F, Staebler A, Wallwiener M (2014). Prognostic relevance of induced and spontaneous apoptosis of disseminated tumor cells in primary breast cancer patients. BMC Cancer..

[24] Schaller G, Fuchs I, Pritze W, Ebert A, Herbst H, Pantel K (1996). Elevated keratin 18 protein expression indicates a favorable prognosis in patients with breast cancer. Clin Cancer Res..

[25] Woelfle U, Sauter G, Santjer S, Brakenhoff R, Pantel K (2004). Down-Regulated Expression of Cytokeratin 18 Promotes Progression of Human Breast Cancer. Clinical Cancer Research..

